# Self-assessment of competences and their impact on the perceived chances for a successful university-to-work transition: the example of tourism degree students in Poland

**DOI:** 10.1007/s11233-021-09081-5

**Published:** 2021-10-27

**Authors:** Danuta Piróg, Wioletta Kilar, Renata Rettinger

**Affiliations:** Uniwersytet Pedagogiczny im Komisji Edukacji Narodowej w Krakowie, ul. Podchorazych 2, 30-084 Kraków, Poland

**Keywords:** Competences, Graduate, Labour market, Principal component analysis (PCA), Poland, Self-assessment, Tourism, University, transition

## Abstract

Competences are the most important career capital a university graduate can have. The objective of the paper is to determine which competences acquired at tourism degree programmes affect students’ self-assessment regarding their own competitive advantage on the labour market. The data was collected during a nationwide diagnostic survey (N = 476) carried out at ten Polish universities among students finishing their tourism degree courses. Principal component analysis allowed us to identify a set of competences that have a significant impact on the students’ perception of their chances of finding employment in the tourism industry. These are: ability to cope with challenges and stress; writing and speaking skills in a foreign language; public speaking; planning and implementation of subject-specific projects; ability to conduct subject-specific research and perseverance. The higher the assessment of each of the above, the more confident of their competitive advantage on the labour market the students were.

## Highlights

- Self-assessment of competences credibly corresponds to the students' perception of their career potential
- Competences are the fundamental career capital of recent university graduates
- PCA helps identify a set of competences that determine self-perception of one’s competitive advantage on the labour market
- Seven competences determine how students of tourism degree courses assess their chances on the Polish labour market
- The topic of tourism degree graduates’ competences in relation to their situation on the labour market is still an under-researched area

## Introduction

The modern labour market has a growing awareness regarding the importance of an individual’s competences in the process of setting up a company and ensuring its dynamic growth (Baum, 2015; Solnet et al., 2012). Employee competences are considered the most important determinant of competitiveness among job seekers and crucial career capital in many sectors of the economy (e.g.: Adeyinka-Ojo, 2018; Iorgulescu & Tăpescu, 2016; López-Bonilla & López-Bonilla, 2014). The significance of competences on the labour market is particularly high for recent university graduates (Allen & Van Der Velden, 2005; Piróg, 2016). Understandably, they enter the job market with less professional experience and fewer contacts than people already employed who are looking to change jobs. The main asset of graduates are primarily their skills acquired during their degree courses. Due to the fact that competences can determine success in university-to-work transition, they are called transition capital (Turska, 2014). For this reason competences should be a fundamental contribution that universities make in educating graduates who are competitive on the labour market and stand a high chance of securing a job (Farmaki, 2018; Jurše & Tominc, 2008; Wilton, 2012).

According to the curricula documentation, graduates of each degree programmes should acquire certain skills. The formal assessment of how well they mastered those skills is shown on their higher education diploma and the attached diploma supplement details the achievements of its holder. An informal, but universally recognised relevant tool of measuring acquired competences is a self-assessment performed by each student. Self-assessment reflects one's individual attitude towards their career potential and being competitive on the labour market (Ferris et al., 2010; Judge & Bono, 2001; Lohan & King, 2016; Marsh et al., 2016; O’Leary, 2017; Potgieter, 2012; RAMA & Sarada, 2017). The higher the self-assessment of one’s skill resources, the higher the assessment of one’s self-worth. Consequently, more self-confident students are willing to put in more effort towards achieving their goals, e.g. finding a job (Rothwell et al., 2008; Savickas, 2013). Research suggest that individuals with high self-esteem maintain optimism in the face of failure, which makes future success (and thus future satisfaction) more likely (Varga et al., 2016).

As shown by research conducted over at least twenty years, independent factors (gender, age and learning environments) can impact self-assessment of. (Bleidorn et al., 2016; Habibollah et al., 2009; Helwig & Ruprecht, 2017; Robins et al., 2002; Zeigler-Hill & Myers, 2012). They can differentiate how people view their own labour potential (Monteiro et al., 2016). On the one hand, it has been established that for students, university is the most influential environment (Kuh et al., 2006; Ovbiagbonhia et al., 2019).

Even though the topic of tourism and recreation degree course students’ and graduates’ competences in the context of their employability has been a frequent subject of academic research: “(…) *there is still a need to investigate the issues associated with those competences provided specifically by tourism higher education in greater depth*” (López-Bonilla & López-Bonilla, 2014, p. 313). An under-researched topic is still the relationship between graduates’ self-assessment of their competences and their transition to the labour market. Recently, some attempts have been made in literature to explore this relationship in more depth. They were largely case studies which referred either to the tourism students’ evaluation of the expected level of importance of the particular competencies for their future profession/ employment in the tourism sector (e.g. Donina, 2020; Palenčíková & Repáňová, 2017; Sándorová, 2019; Wakelin-Theron et al., 2019), or to stakeholders' perceptions of the structure, relevance, adequacy and balance of the courses, the quality of lecture delivery and graduate employability (Felisitas, 2012). This relationship, in turn, constitutes a crucial part of the leading paradigm in the theories of career development. According to this paradigm, self-assessment of competences is a crucial component of career competences and a strong predictor of graduates’ success on the labour market (Chang et al., 2014; Fominiene et al., 2015; Wang & Tsai, 2014). The high significance of this component stems from the fact that self-assessment of competences reflects graduates’ confidence about how much they are worth during the job-seeking process which, in turn, translates into being more determined in their efforts (Savickas, 2011, 2013; Spurk & Abele, 2014).

Taking the above mentioned statements as the main framework, our study intends to fill some of these gaps in empirical research and explore whether and, if so, which competences developed during university education shape the perception of graduates’ own chances for a successful transition into the labour market. In the paper, we focus on the relationship between graduates' competences and the transition into the tourism sector of the labour market. The objective is to determine whether, and, if so, how, the assessment of one's own competences acquired or developed during university education by students of tourism courses impacts the evaluation of one's chances on the labour market and thus can determine self-esteem in general and increase one's competitive edge on the job market.

## Competences of tourism degree graduates and the labour market - literature review

The competences of tourism degree graduates in relation to their situation on the labour market has been rarely discussed (Adeyinka-Ojo, 2018). The studies are mainly papers on chosen competences that are discussed either from the point of view of new challenges resulting from the development of the sector or from the perspective of the changing expectations of the employers. Occasional research projects were conducted on the impact of competences on the success of job-seeking efforts. It has not been established so far which competences determine the self-perception of one’s chances on the labour market by students nearing graduation. However, there is a number of premises that suggest there is a specific group of such competences.

For instance, entrepreneurial competences were examined and a significant relationship was established between: innovation, propensity to take risks, entrepreneurial family background and entrepreneurial intention (Gurel et al., 2010). It was demonstrated that entrepreneurial skills are currently especially important for young people who are entering the labour market because they allow them to face the challenges of this highly competitive and demanding industry (Daniel et al., 2017).

It was highlighted that success rate of people interested in working in tourism increases if they have extensive digital competences, as this sector is highly affected by the Internet economy (Minghetti & Buhalis, 2010; Morellato, 2014). The importance of language skills in the tourism sector was also emphasised as was the need to improve language tuition in the course of study (Frydrychova Klimova & Semradova, 2013; Luka et al., 2013), including writing skills (Frydrychova Klimova, 2014). Intercultural skills were also identified as important for people working or planning to work in tourism (Luka et al., 2013). On the other hand, cultural competence was recognised as one which significantly influences the job performance of tour leaders (Tsaur & Tu, 2019).

A set of specific competences was identified that are particularly important nowadays for the tourism industry. Some researchers have outlined the importance of four categories of skills: related to destination stewardship; politics and ethics; enhanced human resources; dynamic business skills (Sheldon et al., 2008). Other scholars have stressed mainly transversal skills (Donina & Luka, 2014; Zehrer & Mössenlechner, 2009), problem-solving skills, solving operational problems (Christou & Sigala, 2001), customer-oriented skills, adequate behaviour in crisis situations (Raybould & Wilkins, 2005) and transferable skills (Dhiman, 2012).

Among the papers on expectations and preferences of employers, researchers looked into generic competences of tourism graduates and their value for tourism employers (Munar & Montaño, 2009). Other scholars identified expectations of Austrian and Greek employers (Tsitskari et al., 2017; Zehrer & Mössenlechner, 2009). In tourism-related fields, Austrian employers valued: communication skills, empathy, motivation, decision-making abilities, planning abilities, and improvisation abilities. Furthermore, Greek employers expect: professional behaviour and development, leadership and influence, problem solving, organization and time management, (inter) personal skills and communication ability (Tsitskari et al., 2017).

As far as the relationship between graduates' competences and the success of university-to-work transition, so far the only topic examined was students’ perception of the abilities and competencies needed for a successful career in tourism, highlighting the extent to which they consider their undergraduate studies helped them develop the considered competencies (Iorgulescu & Tăpescu, 2016). The academic community in the twentieth century established a list of skills considered to be employability skills. These include mainly soft skills, e.g.: communication, teamwork, planning and organising, lifelong learning, self-awareness, entrepreneurship and a modest number of hard skills (technology, workplace related skills) (Adeyinka-Ojo, 2018).

## Data and methods

The primary objective of the study was to diagnose which competences acquired during a degree course in tourism have the greatest impact on the self-assessment of recent graduates' competitive advantage on the labour market.

Therefore, we put forward the following hypotheses, aligned with the discussed literature concerning competences:

- H1: There is a relationship between the assessment of one's competences and self-evaluation of one's situation on the labour market.
- H2: There is a group of competences that significantly determine the assessment of one's chances on the labour market.
- H3: Independent variables: degree cycle, gender, age and place of study significantly affect the self-assessment of the level of acquired competences.
- H4: Independent variables: degree cycle, gender, age and place of study significantly affect the self-assessment of one's chances on the labour market.

In order to achieve the research objective, we collected data during a nationwide study conducted in May and June 2018 in the form of a diagnostic survey. The population chosen for the study comprised students finishing their bachelor's or master's degree programmes in tourism at public universities. During the research they were completing their final modules and after defending their thesis were able to enter the labour market as university degree holders.^1^

Before the start of the study, we sent official letters to the relevant deans in all ten universities offering degree programmes in tourism asking for consent to conduct the research. All universities agreed to take part in the project.

We preceded the main part of the study with a pilot conducted in April 2018 at a Krakow university with a total of 40 participants. Thanks to the pilot study, we fine-tuned the instructions and made the competence description clearer. For instance, after the pilot study we decided to add examples in brackets for three competences for the purpose of clarity. The results of the pilot study were not included in the analyses.

A total of 496 respondents took part in the main study. Then, we verified the reliability of the completed questionnaires and as a result qualified 476 people for further analysis, out of which more than three quarters were women. The majority of the respondents attended bachelor's courses (over 60%). The remaining students were completing their master's programmes. The population age was rather uniform and was comprised mainly of respondents aged 21-25 who accounted over 90% of the entire study group (see Table 1).

**Table 1:** Descriptive characteristics of the surveyed population, data collected between May and June 2018 by the authors

*Gender*		
Female	367	77.10%
Male	109	22.90%
*Education*		
Bachelor	317	66.60%
Master	159	33.40%
*Age (in years)*		
18 – 20	20	4.20
21 – 25	439	92.23
26 - 30	16	3.36
˃ 31	1	0.21

The respondents in the study had to perform a self-assessment of two areas: how well they mastered a variety of competences in the course of study and their chances in competing for a job in the tourism sector. We chose to rely on self-assessment because it is a credible source of knowledge and a recognised diagnostic tool in social sciences (Ferris et al., 2010; Judge & Bono, 2001; RAMA & Sarada, 2017). Self-assessment is highly regarded in such types of research because respondents have access to information about themselves that outside observers may not be aware of. Secondly, such methodology provides the researchers with quantifiable and transferable data that can then be analysed using advanced statistical methods (Allen & Van Der Velden, 2005). Moreover, empirical research that compares self-perceived and objectively measured competences proved that the greatest majority of students tended to assess their skills objectively, not higher and even slightly lower than expected by their teachers and published criteria (Katowa-Mukwato & Banda, 2016). Finally, self-assessment of one’s competences was used successfully in research in tourism, e.g. studies on managerial skills (Mahachi, 2012). The participants evaluated their level of competences on a scale of 1 to 6. The scale is aligned with the grading system used in Polish schools where 1 stands for fail; 2 - pass; 3 - satisfactory; 4- good; 5- very good; 6 - excellent.

The set of assessed competences was created based on: primary sources, i.e. curricula of tourism degree courses in the surveyed HEIs which included the description of a graduate's skills and competences. Second, the list of competences was created based on the review of literature on the topic. The final set of competences is made up of 36 items including generic/transversal and subject-specific competences in line with the categories adopted in literature (López-Bonilla & López-Bonilla, 2014; Munar & Montaño, 2009).

The respondents assessed their situation on the labour market via reference evaluation, i.e. in relation to other students of the same course and at the same universities - their peers; in relation to tourism students from other universities; in relation to students of related degree course, e.g. recreation or hospitality. We used a 3-point scale where 0 stands for - I have no chances to compete, 1 - I have as much chance as my peers, 2 - my chances are better to those of my peers. For further analysis we used an aggregated indicator based on these three evaluations, where each score was assigned a given weight, respectively: 20, 30 and 50.

Self-confidence about one's chances on the labour market is not linked to a single factor or high assessment of a single skill. The key to analyse self-esteem is to take into account the cumulative and simultaneous role of many factors which determine how one views their competitive advantage (e.g.: Correia et al., 2007; Karli, 2016; Rothwell et al., 2008, 2009; Vargas et al., 2018). Although analysing single variables can lead to academically interesting conclusions, it does not allow for a holistic examination of the process and identifying key competences that play a major role in the perception of one's own chances on the labour market. A method best suited to analyse the concurrent impact of several variables on the assessment and perception of one's situation on the labour market is principal component analysis (e.g.: Vargas et al., 2018; Weber et al., 2009, 2013). Therefore - given the formulated research objective - we used PCA in our analysis. Using this tool, we can extract all components that can actually exist in correlations of a given set of variables and at the same time keep as much information as possible from the primary variables. PCA facilitates the identification of key variables which in the case of the present study are the key competences responsible for self-assessment of one's situation on the labour market.

## Results

In total, the surveyed population evaluated their mastery of all their competences at a level close to “good” during their degree (grade 3.90). Competences they considered as mastered best was the ability to work as part of a team as well as openness and perseverance. The lowest scores were assigned to conducting subject-specific research, using subject-specific software, foreign language writing and speaking skills. There were no major deviations in the evaluation of the above skills because the difference between the highest and the lowest grade amounted to only 1.02 p.p.

The self-assessment is somehow affected by the gender of the respondents. Men scored their skills slightly higher and more equally than women. Skills that female respondents evaluated as those they mastered best were intercultural competence and being able to cope with stress (4.44 each). Women thought they were least competent in subject-specific software (3.27). Men assessed their openness, critical thinking, and the ability to evaluate themselves and others as well mastered and felt least competent in their ability to adapt to new situations.

The self-assessment between undergraduate and postgraduate students varied slightly. Surprisingly, bachelor's degree students evaluated their competences higher than those finishing their master's degrees. The former assessed their interpersonal competences higher and the latter thought they mastered multicultural and subject-specific skills best. Bachelor's degree students ranked their ability to cope with stress as their top skill and master's degree students viewed themselves as performing best in being able to work with people with different cultural backgrounds (see appendix 1). We observed no regularities or differences in assessments depending on age or location of the university.

The majority of the respondents (59 %) believed that they are as attractive to employers as graduates of other universities and degree programmes. Just over 20% considered their chances on the labour market to be superior to these of their peers. The remaining respondents positioned themselves very lowly on the market and were of the opinion that their chances on the job market, when compared to other graduates, were next to zero. The greatest diversity in this respect was observed in relation to the university attended. Students from Kielce and Bydgoszcz were least optimistic about their competitive advantage and students from Poznań and Sosnowiec felt strongest in this respect.

Men were more confident regarding their chances to find work than women (difference of 9.4 p.p.). Over one third of male respondents felt they had better chances on the labour market than other students. Half of them thought they were equally attractive to the employers as others and the remaining group believed they were uncompetitive. Among the female respondents nearly 20% believed they had better chances on the labour market than peers and 60% thought their chances were equal when compared to other students.

**Fig. 1:**
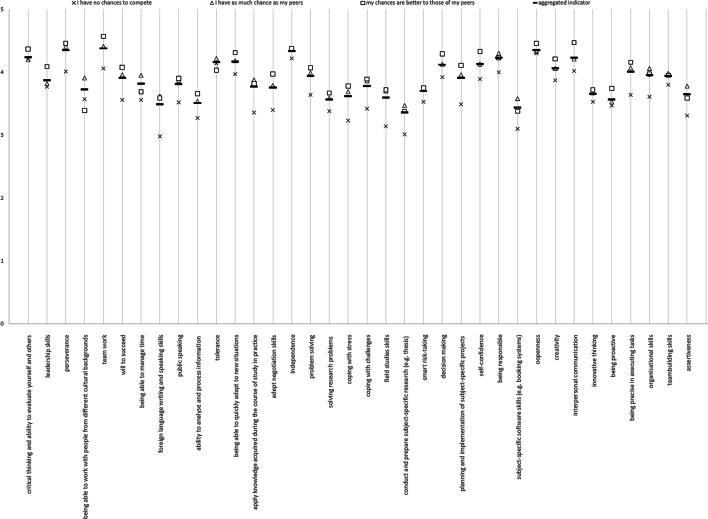
Self-assessment of competences and evaluation of one's chances on the labour market

No significant differences were observed depending on study cycle and respondents' age. The assessment of chances on the labour market in the population of bachelor's and master's degree students differed by no more than 2 p.p. (see Fig. 1).

There is a correlation between the assessment of specific competences and evaluation of one's chances of success on the labour market. As seen in Fig. 1, the higher the assessment of individual skills, the higher the belief in one’s improved chances on the labour market. This relationship applies to 28 out of 36 analysed skills. PCA allows us to conclude which of these competences (based on self-assessment) determine the view on one's competitive advantage on the labour market. Principal component analysis is a statistical procedure aimed, inter alia, at reducing the number of variables that describe a certain phenomenon and revealing the regularities between the variables. The analysis consists of identifying components that are a linear combination of the explored variables. An in-depth analysis of principal components leads to the identification of primary variables that have had a big impact on those principal components. Consequently, the principle component, in which the variance is maximized, becomes representative for this group.

Data collected during the study were normalised and standardised. Principal component analysis of the thirty six studied competences was performed in Statistica software with Varimax normalized rotation. The Cronbach’s alpha and Kaiser–Meyer–Olkin (KMO) were executed to check the reliability of the questionnaire before performing the factor analysis. The higher the internal consistency of the test, the higher the value of these coefficients. Usually values over 0.60 or 0.70 indicate the reliability of a tool. In our study, Cronbach’s coefficient amounted to 0.946, which shows that this set of factors is fully suitable to perform an analysis to extract principal components. KMO amounted to 0.945, which additionally indicates that factor analysis is suitable for this data. The subsequent step was to check the correlation strength between all the variables. It demonstrated a lack of strong correlation between a large number of variables (36) and selected six new factors (principal components) for further study which explain 55.678 % of the total variability. These most important factors were identified based on Keiser’s criterion (factor eigenvalues greater than 1) (see Table 2).

**Table 2:** Results of principal component analysis

Principal components	Eigenvalue	% of the total Variance	Cumulative Eigenvalue	Cumulative %
F1	12.684	35.235	12.685	35.235
F2	2.232	6.202	14.917	41.437
F3	1.609	4.469	16.526	45.906
F4	1.340	3.724	17.867	49.630
F5	1.167	3.241	19.034	52.872
F6	1.010	2.806	20.044	55.678

Note: factor eigenvalues greater than 1

**Fig. 2:**
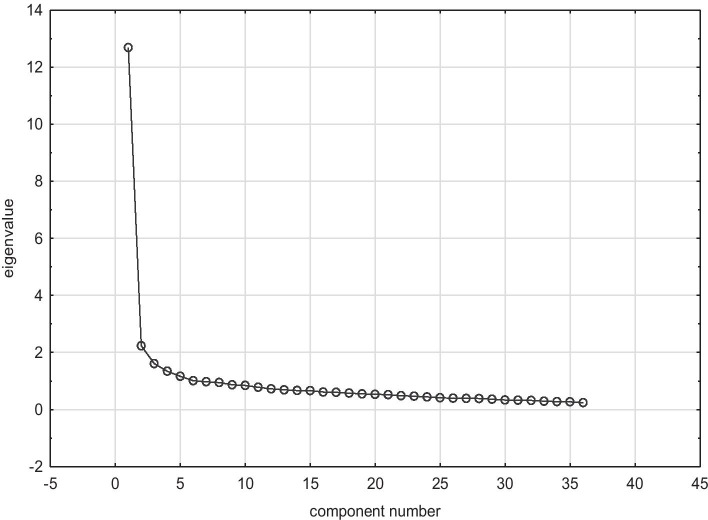
Scree plot

Next, we verified the number of components examining the plot for a visible “kink” thus further reducing the number of components which allowed us to look into these competences that determine the evaluation of one's chances on the labour market among the surveyed students of tourism degree courses. At this stage, the analysis led us to further reduction of the number of competences on the list and we identified 4 or 5 components as being the most important (see Fig. 2).

Next, using the maximum likelihood estimation of eigenvalues^2^ greater than 1.0, we narrowed down the evaluation of one's chances of success on the labour market to three factors.

The values of these three factor coordinates allowed us to finally identify group F1 as the variables/competences that determine the evaluation of one's chances on the labour market. The first factor (F1) represents 33.62% of total variance. It includes competences categorised into: subject-specific, multicultural, personal and cognitive. It has the highest factor loadings with a positive relation with the following seven competences: planning and implementation of subject-specific projects; being able to conduct and prepare subject-specific research (e.g. necessary for a thesis); foreign language writing and speaking skills; public speaking; coping with challenges; coping with stress and perseverance. The analysis showed that the higher the evaluation for each of these, the higher the confidence in one's good standing on the labour market. The highest correlation between the evaluation of one's chances of success on the labour market and self-assessment of one's competences was observed for perseverance (0.628), being able to cope with stress (0.626) and foreign language skills (0.614).

The second factor (F2) - a group of mutually correlated variables which explains 4.66% of total variance comprises the following competences: smart risk-taking; subject-specific software skills; independence; being able to apply knowledge acquired during the course of study in practice; being able to quickly adapt to new situations and working with people with different cultural backgrounds. The third factor (F3), which represents 2.977% of total variance, includes the following five competences: being proactive; innovative thinking; interpersonal communication; creativity; openness. Low or no correlation for variables of the last two factors (2, 3) leads to the conclusion that they are of little significance for the evaluation of one's chances on the labour market and can be disregarded in identifying the competences that determine the view on one's chances of success on the job market (see Tables 3 and 4).

**Table 3:** Summary of principal component analysis –final identification

principal components	Eigenvalue	% of the total variance	Cumulative eigenvalue	Cumulative %
F1	12.10446	33.62349	12.10446	33.62349
F2	1.67951	4.66532	13.78397	38.28881
F3	1.07172	2.97701	14.85569	41.26582

**Table 4:** Results of the PCA showing factor loadings and eigenvalues of each factor

Variable	Factor		
	F1	F2	F3
assertiveness	0.421	0.115	0.339
teambuilding skills	0.374	0.176	0.465
organisational skills	0.410	0.217	0.343
being precise in executing tasks	0.409	0.198	0.324
being proactive	0.281	0.258	0.553
innovative thinking	0.158	0.375	0.617
interpersonal communication	0.239	0.197	0.656
creativity	0.233	0.273	0.628
openness	0.341	0.043	0.590
being responsible	0.535	0.138	0.243
self-confidence	0.535	0.154	0.417
planning and implementation of subject-specific projects	0.586	0.140	0.392
decision making	0.531	0.240	0.215
smart risk-taking	0.132	0.562	0.192
subject-specific software skills (e.g. booking systems)	0.097	0.642	0.194
conduct and prepare subject-specific research (e.g. thesis)	0.561	0.369	0.146
coping with challenges	0.577	0.259	0.124
coping with stress	0.627	0.087	0.282
problem solving	0.519	0.271	0.296
independence	0.207	0.617	0.154
adept negotiation skills	0.384	0.336	0.146
apply knowledge acquired during the course of study in practice (e.g. plan and manage tourist traffic)	0.154	0.716	0.164
being able to quickly adapt to new situations	0.212	0.662	0.154
tolerance	0.326	0.272	0.261
solving research problems	0.271	0.351	0.066
ability to analyse and process information	0.387	0.478	0.187
public speaking	0.595	0.401	0.205
foreign language writing and speaking skills	0.614	0.240	0.247
being able to manage time	0.328	0.298	0.148
field studies skills	0.494	0.317	0.115
will to succeed	0.223	0.564	0.115
team work	0.535	0.291	0.169
being able to work with people from different cultural backgrounds	0.514	0.147	0.296
perseverance	0.628	0.112	0.147
leadership skills	0.529	0.144	0.296
critical thinking and ability to evaluate yourself and others	0.512	0.269	0.185

## Discussion and conclusions

The literature review presented in the paper demonstrates that tourism labour, including competences and their role in a successful transition of graduates into the labour market, remains a relatively minor player in academic research. This research gap becomes even more evident when we compare it to the wider social science arena where academic explorations into these problems are abundant (Ladkin, 2011). Therefore, the paper is an attempt to bridge this gap and verify the formulated hypotheses. Hypothesis 1 was entirely corroborated - there is a link between self-assessment of competences and the evaluation of one's situation on the labour market. The belief in one's competitive advantage in the labour market increased the higher the assessment of one's competences acquired during the degree course. This relationship applied to two thirds of all competences assessed by the respondents.

In line with hypothesis 2, there is a group of competences that significantly determine the assessment of one's chances of success on the labour market. Thanks to the application of principal component analysis, we established that seven competences determine the self-assessment of the chances of success on the labour market performed by students of tourism and recreation degree courses in Poland. We also found out that the better the assessment of their ability to cope with challenges and stress, writing and speaking skills in a foreign language, public speaking, planning and implementation of subject-specific projects, ability to conduct and prepare subject-specific research and perseverance, the better their chances of finding satisfactory employment.

The performed analysis justifies the conclusion that Polish students evaluate their level of mastery of various competences as intermediate and not very varied in relation to other independent variables. The gender and study cycle variables have a medium impact on the self-assessment of competences. Age and location of university are not correlated with these evaluations. This partially corroborates hypothesis 3.

The respondents estimated their competitiveness on the labour market as average (neither superior nor inferior to other graduates). Independent variables only partially shaped the way students viewed their situation on the labour market. One variable that had the greatest impact on how the respondents positioned themselves on the labour market were the location of university and gender. The remaining ones, i.e. cycle of study and age, did not affect the students' self-perception. This means that hypothesis 4 has been corroborated only partially.

Having confronted the results with the current state of research on the topic, we observed a consistent conclusion that competences are the most important career capital of university graduates and that there are varying opinions as to which competences are the key components of this capital. Consequently, the analyses performed in the paper are in parts consistent with the results of previous research on competences, but are also in parts contrary to existing findings.

We have established that a higher assessment of personal skills reinforces the belief in being more attractive to the employer. The significance of these competences for graduates indeed turns out to be highly important for their employability (Adeyinka-Ojo, 2018; Kim & Jeong, 2018).

Self-assessment of one's chances of success on the job market was also shaped by the students’ perceived mastery of foreign languages. Students who evaluated their language skills highly, were also convinced that their chances of finding a job were better when compared to their peers. This finding goes in line with other research on the usefulness of such skills for jobs in the tourism sector (e.g.: Frydrychova Klimova & Semradova, 2013; Tsitskari et al., 2017).

The discrepancy between our results and those of other researchers applies to the evaluation of one's mastery of subject-specific skills in relation to being competitive on the job market. Our analyses demonstrate a high correlation between how students assessed the above competences and their chances of finding employment, i.e. the higher score for the above competences, the higher self-confidence in one's competitive advantage on the labour market. However, the literature review suggests that such competences are the least important for employability in tourism (Adeyinka-Ojo, 2018) and that employers do not value them highly during recruitment (Fallows & Steven, 2000; Zehrer & Mössenlechner, 2009). Such a discrepancy provokes further reflection and an invitation to attempt to understand the reason behind such differences. Polish respondents who self-assessed their subject skills highest were at the same time students who received the top grades from their lecturers during the course of study. Obtaining high grades in specialised and difficult courses are tangible proof that they excelled in meeting academic requirements. This contributed to further reinforcement of their self-esteem, also regarding their situation on the labour market. Additionally, what is crucial is the cultural context and Polish society's attitude towards higher education. Grades at school and university and obtaining a degree are seen as key factors that better position an individual on the job market, even if the significance of grades and a university diploma has slightly diminished in recent years (Piróg, 2015). But it explains the strong correlation between the self-assessment of those skills and one's situation on the job market.

Interestingly, the list of competences that determine students' perceived chances on the labour market does not include digital competences, whereas the literature describes them as being crucial in the tourism sector (e.g.: Morellato, 2014). The reason behind this is that the generation the surveyed students represent acquired these competences before their university education. Currently young people become fluent in technology very early on in their lives and the virtual world is their natural everyday environment (Kachniewska & Para, 2014). This is also why such competences are not viewed as specific career capital but are taken for granted as a basic skill every young person in the twenty-first century should have mastered.

In conclusion of both empirical study results and the literature review, it is worth adding that the COVID-19 pandemic dramatically changed the situation on the tourism labour market all over the world (Nanno, 2020**;** Pham et al., 2021**;** Radlińska, 2020**;** Williams, 2021). These changes are so extensive, profound, both direct and indirect, that their scale and the plethora of implications are impossible to clearly predict at this moment. The repercussions of the pandemic strongly impacted the tourism sector which is manifested both in the decline in output and employment in characteristic tourism industries such as accommodation, restaurants, transportation and in a range of many other industries. The tourism industry needs to comply with the coronavirus-related restrictions of movement which, in turn, generates the need to create new forms of tourism and novel travel products (Pham et al., 2021). Adjusting to the new reality will undoubtedly change the structure of sought-after competences among employees in this sector. The pandemic has created the so-called VUCA environment, which is characterised by volatility, uncertainty, complexity and ambiguity. Therefore, it is expected that the key skills on the VUCA tourism labour market will include, inter alia, self-awareness, flexibility, creativity, critical thinking and problem-solving to serve the industry's evolving needs (Wakelin-Theron et al., 2019). In the light of these changes, the skill set that was identified in the study as significantly determining students' chances of success on the labour market can be universal and useful under any circumstances.

## Limitations

The conducted research project and the presented analyses have some limitations. Despite the fact that the survey was nationwide and included nearly all higher education institutions offering relevant courses and several hundred respondents, the study was conducted in a single country and among tourism students. Therefore, it would be unjustified to draw general conclusions applicable to all graduates in any country and formulate universal statements. It needs to be highlighted that the study was based on self-assessment. Even though it is considered to be a valuable method of measuring competences in academic research, self-assessment carries certain risks. These include inflating or underestimating the scores by some students; however understating one’s assessment has been observed more often than overstating the score (Katowa-Mukwato & Banda, 2016).

The established correlations and facts can be used to illustrate self-assessment of competences in countries with a similar labour market mind-set regarding higher education and other similar conditions governing the general job market situation. It can be assumed with high probability that the defined competence “mix” shapes the perception of one's chances of success on the labour market in the countries of the former socialist bloc and that young people from those countries evaluate their skills in a similar manner. However, because there are no research results of this type in other countries, we can only make predictions but not formulate definitive statements.

Despite the above limitations, the results presented in the paper can be applied on two dimensions – both academic and management. In terms of academic applications, the results provide a solid data set for drawing methodological and subject-specific comparisons, in particular regarding the application of PCA to such analyses for future research projects on a similar topic. In the management dimension, the results provide specialists in tourism-related curriculum design with useful information about which competences are missing and which are developed excessively from the perspective of students nearing graduation. Such information can help modify curricula to facilitate the development of such competences that according to students were not mastered at a satisfactory level.

## Appendix 1

See Table 5.

**Table 5:** Self-assessment of competences

Competence	average	Gender		Degree cycle		Sd
		female	male	Bachelor	Master	
critical thinking and ability to evaluate yourself and others	4.24	4.26	4.17	4.26	4.19	1.19
leadership skills	3.87	3.82	4.05	3.91	3.79	1.33
perseverance	4.35	4.40	4.17	4.35	4.35	1.16
being able to work with people from different cultural backgrounds	4.38	4.44	4.17	4.32	4.48	1.12
team work	3.91	3.93	3.82	3.92	3.88	1.23
will to succeed	3.77	3.75	3.81	3.80	3.70	1.18
field studies skills	3.82	3.86	3.67	3.87	3.72	1.27
being able to manage time	3.73	3.74	3.70	3.79	3.60	1.39
foreign language writing and speaking skills	4.17	4.19	4.10	4.25	4.01	1.18
public speaking	3.94	3.94	3.94	4.01	3.81	1.08
ability to analyse and process information	3.91	3.91	3.90	4.01	3.69	1.08
solving research problems	3.49	3.44	3.67	3.61	3.26	1.30
tolerance	3.81	3.80	3.87	3.85	3.74	1.27
being able to quickly adapt to new situations	3.51	3.50	3.55	3.52	3.50	1.12
apply knowledge acquired during the course of study in practice (e.g. plan and manage tourist traffic)	3.57	3.52	3.72	3.55	3.60	1.25
adept negotiation skills	4.16	4.24	3.90	4.11	4.26	1.32
independence	3.60	3.59	3.62	3.68	3.45	1.30
problem solving	3.76	3.74	3.83	3.82	3.64	1.25
coping with stress	4.34	4.40	4.12	4.37	4.28	1.28
coping with challenges	3.62	3.59	3.73	3.68	3.50	1.37
conduct and prepare subject-specific research (e.g. thesis)	3.78	3.78	3.78	3.82	3.71	1.19
subject-specific software skills (e.g. booking systems)	3.36	3.27	3.66	3.28	3.52	1.32
smart risk-taking	3.44	3.39	3.63	3.41	3.52	1.33
decision making	3.70	3.64	3.88	3.77	3.56	1.28
planning and implementation of subject-specific projects	4.12	4.14	4.06	4.15	4.08	1.14
self-confidence	4.13	4.11	4.18	4.12	4.16	1.22
being responsible	4.23	4.34	3.86	4.21	4.26	1.18
openness	4.35	4.39	4.22	4.31	4.44	1.25
creativity	4.06	4.06	4.06	4.10	3.98	1.19
interpersonal communication	4.23	4.25	4.15	4.21	4.26	1.13
innovative thinking	3.66	3.66	3.63	3.71	3.56	1.17
being proactive	3.57	3.57	3.57	3.62	3.47	1.06
being precise in executing tasks	4.01	4.07	3.82	3.97	4.09	1.12
organisational skills	3.96	4.04	3.69	3.89	4.08	1.22
teambuilding skills	3.94	3.93	3.95	3.83	4.15	1.13
assertiveness	3.65	3.67	3.58	3.62	3.71	1.26

Note: Grading scale: 1 – unsatisfactory; 2 – acceptable; 3 – satisfactory; 4 – good; 5 – very good; 6 – excellent

## Footnotes

1 In 2018 degree programmes in tourism and recreation were offered in Poland by ten universities and 12 other higher education institutions (e.g. economic HEIs or academies of sport education) https://polon.nauka.gov.pl/opi/aa/rejestry/szkolnictwo?execution = e3s1). These were: the University of Szczecin, Pomeranian University in Słupsk, the Adam Mickiewicz University in Poznań, University of Krakow, the University of Lodz, the Jan Kochanowski University in Kielce, the Nicolaus Copernicus University in Toruń, Maria Curie-Skłodowska University in Lublin, the University of Silesia, Kazimierz Wielki University in Bydgoszcz.

2 Eigenvalue **–** in factor analysis it is a measure that informs how much more variance is explained by a given factor compared to variance explained by a single variable

## References

[CR1] Adeyinka-Ojo, S. (2018). A strategic framework for analysing employability skills deficits in rural hospitality and tourism destinations. *Tourism Management Perspectives, 27*, 47–54. 10.1016/j.tmp.2018.04.005

[CR2] Allen, J., & Van Der Velden, R. (2005). The role of self-assessment in measuring skills. *REFLEX Working Paper Series, 2*, 1–25 http://www.fdewb.unimaas.nl/roa/reflex/publicationspublic.htm

[CR3] Baum, T. (2015). Human resources in tourism: Still waiting for change? - a 2015 reprise. *Tourism Management, 50*, 204–212. 10.1016/j.tourman.2015.02.001

[CR4] Bleidorn, W., Arslan, R. C., Denissen, J. J. A., Rentfrow, P. J., Gebauer, J. E., Potter, J., & Gosling, S. D. (2016). Age and gender differences in self-esteem - a cross-cultural window. *Journal of Personality and Social Psychology, 111*(3), 396–410. 10.1037/pspp0000078.supp 26692356 10.1037/pspp0000078

[CR5] Chang, H. T., Feng, C. Y., & Shyu, C. L. (2014). Individual management and counseling as moderators in achieving career competencies and success. *Social Behavior and Personality, 42*(5), 869–880. 10.2224/sbp.2014.42.5.869

[CR6] Christou, E., & Sigala, M. (2001). Professional development in hospitality and tourism education: A strategy for the 21st century. *The International Journal of Tourism Research, 3*(4), 328–330.

[CR7] Correia, A., Moço, C., & Oom do Valle, P. (2007). Modeling motivations and perceptions of Portuguese tourists. *Journal of Business Research, 60*(1), 76–80. 10.1016/j.jbusres.2005.10.013

[CR8] Daniel, A. D., Costa, R. A., Pita, M., & Costa, C. (2017). Tourism education: What about entrepreneurial skills? *Journal of Hospitality and Tourism Management, 30*, 65–72. 10.1016/j.jhtm.2017.01.002

[CR9] Dhiman, M. C. (2012). Employers’ perceptions about tourism management employability skills. *Anatolia. An International Journal of Tourism and Hospitality Research, 23*(3), 359–372. 10.1080/13032917.2012.711249

[CR10] Donina, A. (2020). *Development of professional competence in higher education topical for the tourism. Summary of the Doctoral Thesis*. RTU Press.

[CR11] Donina, A., & Luka, I. (2014). The compliance of tourism education with industry needs in Latvia. *European Journal of Tourism, Hospitality and Recreation, 102*(15), 2013.

[CR12] Fallows, S., & Steven, C. (2000). Building employability skills into the higher education curriculum: A university-wide initiative. *Education + Training, 42*(2), 75–83. 10.1108/00400910010331620

[CR13] Farmaki, A. (2018). Tourism and hospitality internships: A prologue to career intentions? *Journal of Hospitality, Leisure, Sport and Tourism Education, 23*, 50–58. 10.1016/j.jhlste.2018.06.002

[CR14] Felisitas, C. (2012). The hospitality and tourism honours degree programme: Stakeholders perceptions on competencies developed. *Journal of Hospitality Management and Tourism, 3*(1), 12–22. 10.5897/jhmt11.025

[CR15] Ferris, D. L., Lian, H., Brown, D. J., Pang, F. X. J., & Keeping, L. M. (2010). Self-esteem and job performance: The moderating role of self-esteem contingencies. *Personnel Psychology, 63*(3), 561–593. 10.1111/j.1744-6570.2010.01181.x

[CR16] Fominiene, V. B., Mejeryte-Narkeviciene, K., & Wozniewicz-Dobrzynska, M. (2015). Employees’career competence for career success: Aspect of human resources management in tourism sector. *Transformations in Business & Economics, 14*(2b), 481–493.

[CR17] Frydrychova Klimova, B. (2014). Students of Management of Tourism and their writing competences. *Procedia - Social and Behavioral Sciences, 122*, 438–442. 10.1016/j.sbspro.2014.01.1368

[CR18] Frydrychova Klimova, B., & Semradova, I. (2013). Developing language competences for management tourism students. *Procedia - Social and Behavioral Sciences, 93*, 517–521. 10.1016/j.sbspro.2013.09.231

[CR19] Gurel, E., Altinay, L., & Daniele, R. (2010). Tourism students’ entrepreneurial intentions. *Annals of Tourism Research, 37*(3), 646–669. 10.1016/j.annals.2009.12.003

[CR20] Habibollah, N., Tengku Aizan, H., Jamaluddin, S., Rohani, A., & Kumar, V. (2009). Self esteem, gender and academic achievement of undergraduate students. *American Journal of Scientific Research, 3*, 26–37.

[CR21] Helwig, N. E., & Ruprecht, M. R. (2017). Age, gender, and self-esteem: A sociocultural look through a nonparametric lens. *Archives of Scientific Psychology, 5*(1), 19–31. 10.1037/arc0000032

[CR22] Iorgulescu, M.-C., & Tăpescu, I. A. (2016). Business and tourism students’ considerations on future career. In M. H. Bilgin & H. Danis (Eds.), *Entrepreneurship, business and economics - Vol. 1 proceedings of the 15th Eurasia business and economics society conference* (pp. 183–200). Springer. 10.1007/978-3-319-27570-3_16

[CR23] Judge, T. A., & Bono, J. E. (2001). Relationship of core self-evaluations traits - self-esteem, generalized self-efficacy, locus of control, and emotional stability - with job satisfaction and job performance: A meta-analysis. *Journal of Applied Psychology, 86*(1), 80–92. 10.1037/0021-9010.86.1.80 11302235 10.1037/0021-9010.86.1.80

[CR24] Jurše, M., & Tominc, P. (2008). Professional competences of graduates as a labour market mechanism for aligning business school curriculum reform with the Bologna declaration principles. *Journal of Contemporary Management Issues, 13*(1), 17–36.

[CR25] Kachniewska, M., & Para, A. (2014). Pokolenie Y na turystycznym rynku pracy: fakty, mity i wyzwania. *Rozprawy Naukowe Akademii Wychowania Fizycznego We Wrocławiu, 45*, 153–166.

[CR26] Karli, U. (2016). Adaptation and validation of self-perceived employability scale: An analysis of sports department students and graduates. *Educational Research and Reviews, 11*(8), 848–859. 10.5897/ERR2016.2712

[CR27] Katowa-Mukwato, P., & Banda, S. S. (2016). Self-perceived versus objectively measured competence in performing clinical practical procedures by final year medical students. *International Journal of Medical Education, 7*, 122–129. 10.5116/ijme.5709.2a7e 27132255 10.5116/ijme.5709.2a7e PMC4860286

[CR28] Kim, H. J., & Jeong, M. (2018). Research on hospitality and tourism education: Now and future. *Tourism Management Perspectives, 25*, 119–122. 10.1016/j.tmp.2017.11.025

[CR29] Kuh, G. D., Kinzie, J., Buckley, J. A., Bridges, B. K., & Hayek, J. C. (2006). What Matters to Student Success : A Review of the Literature. In *Commissioned Report for the National Symposium on Postsecondary Student Success Spearheading a Dialog on Student Success*. https://nces.ed.gov/npec/papers.asp.

[CR30] Ladkin, A. (2011). Exploring tourism labor. *Annals of Tourism Research, 38*(3), 1135–1155. 10.1016/j.annals.2011.03.010

[CR31] Lohan, A., & King, F. (2016). Self-esteem: Defining, measuring and promoting an elusive concept. *REACH - Journal of Special Needs Education in Ireland, 29*(2), 116–127.

[CR32] López-Bonilla, J. M., & López-Bonilla, L. M. (2014). Holistic competence approach in tourism higher education: An exploratory study in Spain. *Current Issues in Tourism, 17*(4), 312–326. 10.1080/13683500.2012.720248

[CR33] Luka, I., Vaidesvarans, S., & Vinklere, D. (2013). Educating tourism students for work in a multicultural environment. *Journal of Teaching in Travel and Tourism, 13*(1), 1–29. 10.1080/15313220.2012.729448

[CR34] Mahachi, D. (2012). Students’ perceptions of managerial competencies: A study of undergraduate tourism and hospitality students at the University of Botswana. *Journal of Human Resources in Hospitality and Tourism, 11*(3), 239–258. 10.1080/15332845.2012.673087

[CR35] Marsh, H. W., Martin, A. J., Yeung, A., & Craven, R. (2016). Competence self- perceptions. In A. J. Elliot, C. Dweck, & D. Yeager (Eds.), *Handbook of competence and motivation*. Guilford Press.

[CR36] Minghetti, V., & Buhalis, D. (2010). Digital Divide in Tourism. *Journal of Travel Research, 49*(3), 267–281. 10.1177/0047287509346843

[CR37] Monteiro, S., Almeida, L., & Aracil, A. G. (2016). Graduates’ perceptions of competencies and preparation for labour market transition: The effect of gender and work experience during higher education. *Higher Education, Skills and Work-Based Learning, 6*(2), 208–220. 10.1108/HESWBL-09-2015-0048

[CR38] Morellato, M. (2014). Digital competence in tourism education: Cooperative-experiential learning. *Journal of Teaching in Travel and Tourism, 14*(2), 184–209. 10.1080/15313220.2014.907959

[CR39] Munar, A. M., & Montaño, J. J. (2009). Generic competences and tourism graduates. *Journal of Hospitality, Leisure, Sport and Tourism Education, 8*(1), 70–84. 10.3794/johlste.81.206

[CR40] Nanno, M. (coord. (2020). The impact of the COVID-19 pandemic on the tourism sector in Latin America and the Caribbean , and options for a sustainable and resilient recovery Thank you for your interest in this ECLAC publication. In *International Trade series* (Vol. 157, Issue LC/TS.2020/147). https://repositorio.cepal.org/handle/11362/46502

[CR41] O’Leary, S. (2017). Graduates’ experiences of, and attitudes towards, the inclusion of employability-related support in undergraduate degree programmes; trends and variations by subject discipline and gender. *Journal of Education and Work, 30*(1), 84–105. 10.1080/13639080.2015.1122181

[CR42] Ovbiagbonhia, A. R., Kollöffel, B., & den Brok, P. (2019). Educating for innovation: Students’ perceptions of the learning environment and of their own innovation competence. *Learning Environments Research, 22*(3), 387–407. 10.1007/s10984-019-09280-3

[CR43] Palenčíková, Z., & Repáňová, T. (2017). Professional competencies of tourism graduates. *Economic Theory and Practice ETAP 2017*

[CR44] Pham, T. D., Dwyer, L., Su, J. J., & Ngo, T. (2021). COVID-19 impacts of inbound tourism on Australian economy. *Annals of Tourism Research, 88*(103179). 10.1016/j.annals.2021.103179 10.1016/j.annals.2021.103179 PMC9754951 36540369

[CR45] Piróg, D. (2015). *Przechodzenie absolwentów studiów geograficznych na rynek pracy: proces, determinanty, predykcja*. Wydawnictwo Naukowe Uniwersytetu Pedagogicznego.

[CR46] Piróg, D. (2016). The role of competences for geography higher education in university-to-work transition. *Geographia Polonica, 89*(2), 233–248. 10.7163/GPol.0055

[CR47] Potgieter, I. (2012). The relationship between the self-esteem and employability attributes of postgraduate business management students. *SA Journal of Human Resource Management, 10*(2), 1–15. 10.4102/sajhrm.v10i2.419

[CR48] Radlińska, K. (2020). Pandemia COVID-19 Implikacje dla polskiego rynku pracy. *Zeszyty Naukowe Wydziału Nauk Ekonomicznych, Politechnika Koszalińska, 24*, 113–126.

[CR49] RAMA, L., & Sarada, S. (2017). Role of self-esteem and self-efficacy on competence - a conceptual framework. *IOSR Journal of Humanities and Social Science, 22*(2), 33–39. 10.9790/0837-2202053339

[CR50] Raybould, M., & Wilkins, H. (2005). Over qualified and under experienced: Turning graduates into hospitality managers. *International Journal of Contemporary Hospitality Management, 17*(3), 203–216. 10.1108/09596110510591891

[CR51] Robins, R. W., Trzesniewski, K. H., Tracy, J. L., Gosling, S. D., & Potter, J. (2002). Global self-esteem across the life span. *Psychology and Aging, 17*(3), 423–434. 12243384

[CR52] Rothwell, A., Herbert, I., & Rothwell, F. (2008). Self-perceived employability: Construction and initial validation of a scale for university students. *Journal of Vocational Behavior, 73*(1), 1–12. 10.1016/j.jvb.2007.12.001

[CR53] Rothwell, A., Jewell, S., & Hardie, M. (2009). Self-perceived employability: Investigating the responses of post-graduate students. *Journal of Vocational Behavior, 75*(2), 152–161. 10.1016/j.jvb.2009.05.002

[CR54] Sándorová, Z. (2019). The importance of intercultural communicative competences for tourism labour market: Students’ views and their self-assessment. *Journal of Language and Cultural Education, 7*(1), 103–117. 10.2478/jolace-2019-0007

[CR55] Savickas, M. L. (2011). New questions for vocational psychology: Premises, paradigms, and practices. *Journal of Career Assessment, 19*(3), 251–258. 10.1177/1069072710395532

[CR56] Savickas, M. L. (2013). Career construction theory and practice. In S. D. Brown & R. W. Lent (Eds.), *Career development and counseling: Putting theory and research to work* (Second ed., pp. 147–183). John Wiley & Sons, Inc.

[CR57] Sheldon, P., Fesenmaier, D., Woeber, K., Cooper, C., & Antonioli, M. (2008). Tourism education futures, 2010–2030: Building the capacity to Lead. *Journal of Teaching in Travel & Tourism, 7*(3), 61–68. 10.1080/15313220801909445

[CR58] Solnet, D., Kralj, A., & Kandampully, J. (2012). Generation Y employees: An examination of work attitude differences. *Journal of Applied Management and Entrepreneurship, 17*(3), 36–54.

[CR59] Spurk, D., & Abele, A. E. (2014). Synchronous and time-lagged effects between occupational self-efficacy and objective and subjective career success: Findings from a four-wave and 9-year longitudinal study. *Journal of Vocational Behavior, 84*(2), 119–132. 10.1016/j.jvb.2013.12.002

[CR60] Tsaur, S. H., & Tu, J. H. (2019). Cultural competence for tour leaders: Scale development and validation. *Tourism Management, 71*, 9–17. 10.1016/j.tourman.2018.09.017

[CR61] Tsitskari, E., Goudas, M., Tsalouchou, E., & Michalopoulou, M. (2017). Employers’ expectations of the employability skills needed in the sport and recreation environment. *Journal of Hospitality, Leisure, Sport and Tourism Education, 20*, 1–9. 10.1016/j.jhlste.2016.11.002

[CR62] Turska, E. (2014). *Kapitał kariery ludzi młodych: Uwarunkowania i konsekwencje*. Wydawnictwo Uniwersytetu Śląskiego.

[CR63] Varga, E., Szira, Z., Bárdos, K. I., & Hajós, L. (2016). The most relevant labour market competencies for employers and their assessment by students. *Practice and Theory in Systems of Education, 11*(2), 95–104. 10.1515/ptse-2016-0012

[CR64] Vargas, R., Sánchez-Queija, M. I., Rothwell, A., & Parra, Á. (2018). Self-perceived employability in Spain. *Education + Training, 60*(3), 226–237. 10.1108/ET-03-2017-0037

[CR65] Wakelin-Theron, N., Ukpere, W. I., & Spowart, J. (2019). Determining tourism graduate employability, knowledge, skills, and competencies in a VUCA world: Constructing a tourism employability model. *African Journal of Hospitality, Tourism and Leisure, 8*(3), 1–18.

[CR66] Wang, Y.-F., & Tsai, C.-T. (. S.). (2014). Employability of hospitality graduates: Student and industry perspectives. *Journal of Hospitality & Tourism Education, 26*(3), 125–135. 10.1080/10963758.2014.935221

[CR67] Weber, M. R., Crawford, A., & Lee, J. (Jay), & Dennison, D. (2013). An exploratory analysis of soft skill competencies needed for the hospitality industry. *Journal of Human Resources in Hospitality and Tourism, 12*(4), 313–332. 10.1080/15332845.2013.790245

[CR68] Weber, M. R., Finley, D. A., Crawford, A., & Rivera, D. (2009). An exploratory study identifying soft skill competencies in entry-level managers. *Tourism and Hospitality Research, 9*(4), 353–361. 10.1057/thr.2009.22

[CR69] Williams, C. C. (2021). Impacts of the coronavirus pandemic on Europe’s tourism industry: Addressing tourism enterprises and workers in the undeclared economy. *International Journal of Tourism Research, 23*(1), 79–88. 10.1002/jtr.2395

[CR70] Wilton, N. (2012). The impact of work placements on skills development and career outcomes for business and management graduates. *Studies in Higher Education, 37*(5), 603–620. 10.1080/03075079.2010.532548

[CR71] Zehrer, A., & Mössenlechner, C. (2009). Key competencies of tourism graduates: The employers’ point of view. *Journal of Teaching in Travel and Tourism, 9*(3–4), 266–287. 10.1080/15313220903445215

[CR72] Zeigler-Hill, V., & Myers, E. M. (2012). A review of gender differences in self-esteem. In S. P. McGeown (Ed.), *Psychology research progress. Psychology of gender differences (pp. 131–143)*. Nova Science Publishers.

